# Isolation and Structural Characterization of Melanins from Red and Yellow Varieties of *Stropharia rugosoannulata*

**DOI:** 10.3390/ijms26146985

**Published:** 2025-07-21

**Authors:** Zhen-Fei Xie, Wei-Wei Zhang, Shun-Yin Zhao, Xiao-Han Zhang, Shu-Ning You, Chun-Mei Liu, Guo-Qing Zhang

**Affiliations:** 1 College of Plant Science and Technology, Beijing University of Agriculture, Beijing 102206, China; 202230212051@bua.edu.cn (Z.-F.X.); wwzhang@bua.edu.cn (W.-W.Z.); 202230212071@bua.edu.cn (S.-Y.Z.); zhangxiaohan000910@163.com (X.-H.Z.); y741690177@126.com (S.-N.Y.); 2 College of Bioscience and Resources Environment, Beijing University of Agriculture, Beijing 102206, China

**Keywords:** cap color, chemical composition, melanin extraction, ultrastructural characteristics

## Abstract

Melanin is a complex natural pigment that imparts a variety of colors to the fruiting bodies of edible fungi, influencing both their nutritional quality and commercial value. *Stropharia rugosoannulata* is an emerging type of edible fungus that has been widely cultivated in recent years. It can be categorized into red and yellow varieties based on cap color, while its pigment characteristics remain unclear. In this study, the melanins from the two varieties were obtained using an alkaline extraction and acid precipitation method, followed by comprehensive characterization of their chemical properties and ultrastructural features. Both melanins displayed distinct absorption maxima at approximately 211 nm. The melanin extracted from the red variety consisted of 55.63% carbon (C), 7.40% hydrogen (H), 30.23% oxygen (O), 5.99% nitrogen (N), and 0.64% sulfur (S), whereas the yellow variety comprised 52.22% C, 6.74% H, 29.70% O, 5.91% N, and 0.99% S. Both types of melanin included eumelanin and phaeomelanin forms, with eumelanin being the predominant type. Variations in the quantities and relative proportions of eumelanin and phaeomelanin contributed to the observed color differences in the mushroom caps. Ultrastructural micrographs revealed the melanins were primarily localized in the cell wall, consistent with findings in other fungal species. These findings contribute valuable insights into fundamental knowledge and potential applications of mushroom pigments.

## 1. Introduction

Natural pigments are widely distributed in plants, animals, and microorganisms, and have diverse applications in food, cosmetics, medicine, and textiles [1,2,3]. In contrast to synthetic pigments, natural pigments are not only more environmentally sustainable but also exhibit a wide range of biological activities, including antitumor and antioxidant properties, which have contributed to their growing prominence in the global market [4,5]. Furthermore, microbial pigments offer distinct advantages over natural pigments from other sources in terms of yield, sustainable supply, cost, and stability [6]. Based on their structural characteristics, microbial pigments can be categorized into several classes, including carotenoids, melanins, azaphilones, anthraquinones, etc. [7,8]. Among these, melanins represent the most prevalent class of natural pigments derived from microorganisms. They play a pivotal role in microbial responses to environmental stressors and exhibit a variety of beneficial properties, including hypolipidemic, hepatoprotective, antitumor, and antioxidant activities [9,10].

Melanin, first identified in 1840 as a dark pigment derived from eye membranes [10], has been extensively characterized as a versatile pigment exhibiting a broad spectral range from black and brown to yellow and red [11,12]. Structurally, melanins represent a class of intricate heterogeneous biopolymers predominantly biosynthesized through the polymerization of indole and its derivatives [9,11]. These biopolymers are systematically categorized into three principal types based on their monomeric composition: eumelanin, pheomelanin, and allomelanin. Eumelanin and allomelanin typically demonstrate black to brown pigmentation, whereas pheomelanin is characterized by its distinctive red to yellow coloration [10]. This structural diversity is particularly evident in mushrooms, where the distinct cap pigmentation patterns (ranging from black to yellow and pink) observed in various *Pleurotus* species have been chemically characterized as combinatorial mixtures of eumelanin and pheomelanin [13]. To date, melanin pigments have been identified and characterized in numerous mushroom species, including but not limited to *Agaricus bisporus* [14], *Armillaria cepistipes* [15], *Auricularia auricula* [16], *Chroogomphus rutilus* [17], *Lentinula edodes* [18], *Morchella sextelata* [19] and *Ophiocordyceps sinensis* [20]. This widespread distribution of melanin across taxonomically diverse fungal species underscores its fundamental biological significance and highlights its promising potential in the kingdom Fungi.

*Stropharia rugosoannulata*, commonly known as the wine cap mushroom, is classified within the phylum Basidiomycota, class Agaricomycetes, order Agaricales, family Strophariaceae, and genus *Stropharia* [21]. This species has garnered significant attention in China due to its remarkable lignocellulosic decomposition capacity and efficient utilization of agricultural and forestry wastes, such as wood chips and crop straws [22,23]. Characterized by straightforward cultivation techniques and substantial economic and ecological benefits, *S. rugosoannulata* has seen widespread adoption across the country in recent years. According to the latest statistical data from the China Edible Fungi Association, the national production of *S. rugosoannulata* reached 493,800 tons in 2023, reflecting a notable increase 20.29% compared to the previous year (2022). Additionally, *S. rugosoannulata* is rich in active components such as polysaccharides and umami peptides, which exhibit various bioactivities, including antioxidant, antitumor, and hypoglycemic effects [24,25,26]. The species exhibits two primary cultivated varieties characterized by distinct cap coloration: red and yellow phenotypes. However, under practical cultivation conditions, the pigmentation of fruiting bodies demonstrates significant phenotypic plasticity, with cap colors ranging from red and yellow to grayish-white and white, depending on the ambient light intensity during development. This chromatic variation may be attributed to the differential expression of pigment biosynthesis pathways in response to environmental light stimuli. Despite its phenotypic significance, the pigmentation mechanisms and corresponding chemical composition in *S. rugosoannulata* remain inadequately characterized, with a paucity of scientific literature addressing this critical aspect of its biology.

Although many edible fungi, such as *A. bisporus*, *L. edodes*, and *M. sextelata*, have been reported to contain melanins, there is a lack of systematic research on the pigments of edible fungi. Additionally, various pigments, such as *β*-carotene, azaphilones, and flavins, are also present in fungi [7]. *S. rugosoannulata* primarily exhibits red and yellow colors in its cap, but research on its cap pigments is limited. Wang et al. [27] isolated anthocyanin pigments from *S. rugosoannulata*, which marks the first report of anthocyanins in edible fungi. In our preliminary experiments, we initially isolated melanin components from the pileipellis of *S. rugosoannulata* using an alkaline extraction and acid precipitation method. Based on previous research on pigments in edible fungi, we tentatively speculate that melanin may be an important pigment component of *S. rugosoannulata*. Therefore, in this study, pigments were extracted and identified from *S. rugosoannulata* exhibiting distinct red and yellow cap colors. The physicochemical properties and microscopic morphology of these pigments were investigated in detail. These findings will substantially enhance our understanding of the biological foundations of *S. rugosoannulata* and the complex mechanisms underlying fungal pigmentation.

## 2. Results and Discussion

### 2.1. Isolation of the Pigments

**Figure 1:**
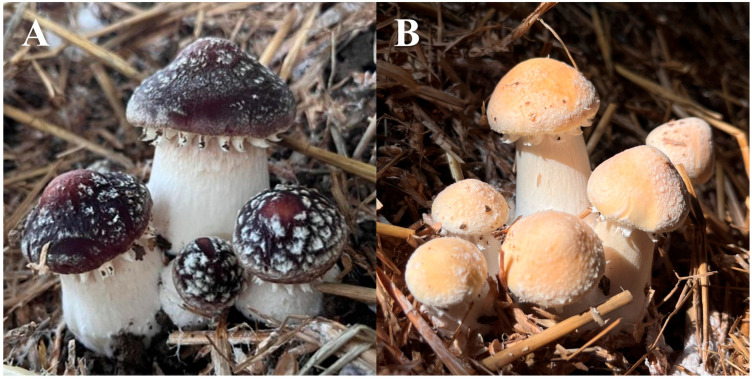
Morphological characteristics of the near-mature mushroom fruiting bodies (not yet opened their caps) in the red (**A**) and yellow (**B**) varieties of *S. rugosoannulata*.

Through repeated cycles of alkaline extraction, acid precipitation, organic solvent washing, and freeze-drying, we successfully isolated the pigments from the pileipellis of the red and yellow varieties of *S. rugosoannulata*. The two isolated pigments from red and yellow varieties were observed to be black and yellowish brown, respectively. The final yields of the pigments were 4.0% and 3.5%, respectively, corresponding to 40 mg and 35 mg of purified pigments obtained from 1.0 g of freeze-dried pileipellis powder. Previous studies reported pigment recovery rates of 0.61% to 1.00% from the caps of three species of fresh *Pleurotus* spp. [13], while the pigment content in the cap of *M. sextelata* was found to be 2.82 mg/g (obtained from dried cap powder) [19]. In contrast to oyster mushrooms and morels, the pigments in *S. rugosoannulata* are exclusively localized in the pileipellis tissue of the cap (Figure 1). Using whole cap as extraction material would result in a high proportion of non-pigment components, significantly affecting the extraction efficiency and recovery rate of the pigments. Therefore, in our pigment extraction protocol for *S. rugosoannulata*, we specifically selected pileipellis tissues from representative red and yellow varieties, resulting in higher recovery rates compared to those obtained from oyster mushrooms and morels.

### 2.2. High-Performance Liquid Chromatography (HPLC) Analysis

**Figure 2:**
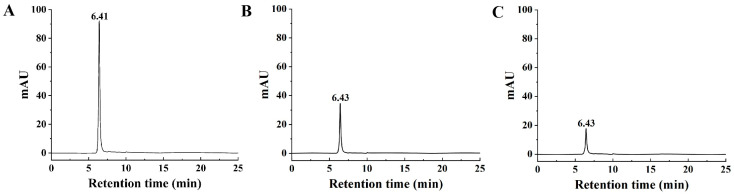
HPLC chromatograms of standard eumelanin (**A**) and isolated melanins from red (**B**) and yellow (**C**) varieties of *S. rugosoannulata*.

The HPLC chromatograms of the isolated melanins and standard eumelanin are presented in Figure 2. The chromatographic profile of the standard eumelanin displayed a single, symmetrical elution peak with a retention time of 6.41 min (Figure 2A). Similarly, both isolated melanins exhibited single, symmetrical elution peaks with retention times of 6.43 min (Figure 2B,C). The HPLC chromatograms of the two isolated melanins showed a single peak analogous to that of the standard eumelanin. These results indicate that high-purity pigments from the pileipellis of *S. rugosoannulata*, primarily composed of eumelanin, can be obtained through multiple repetitions of the alkaline extraction and acid precipitation processes. This indicates that melanin, particularly eumelanin, is the principal component of the pigments derived from the pileipellis of *S. rugosoannulata*, consistent with previous reports on melanins from *M. sextelata* [19] and *Pleurotus* spp. [13]. Furthermore, Wang et al. [27] reported the presence of anthocyanins from the pileipellis of *Stropharia rugosoannulata*, marking the first identification of anthocyanins in edible fungi. This suggests that the pileus of *S. rugosoannulata* may contain multiple types of pigments.

### 2.3. Chemical Composition Analysis of the Pigments

#### 2.3.1. UV–Visible Spectra

**Figure 3:**
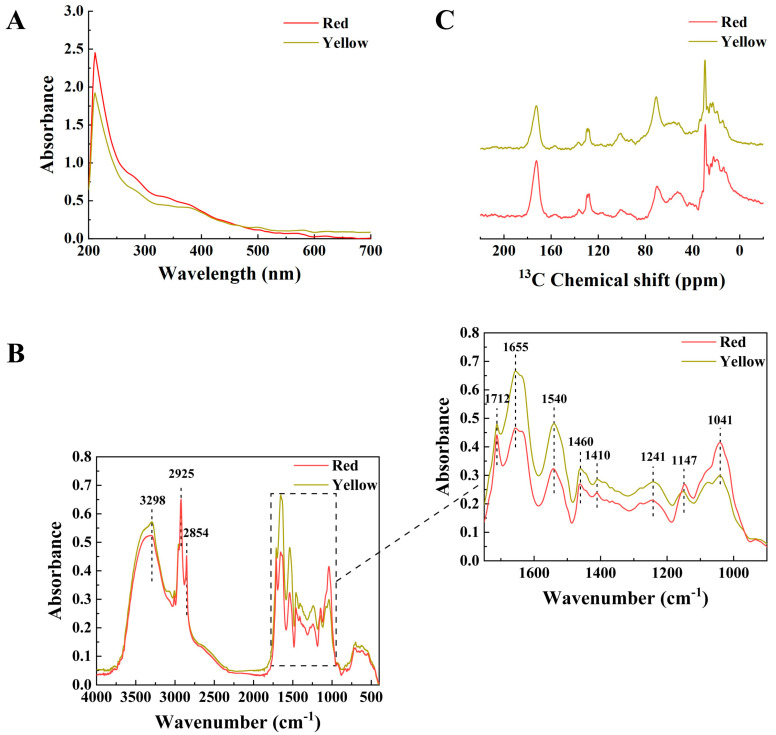
Spectral characteristics of the pigments isolated from red and yellow varieties of *S. rugosoannulata*. (**A**) UV–visible spectra; (**B**) FTIR spectra; (**C**) NMR spectra.

The UV–visible light absorption characteristics of the two isolated pigments are presented in Figure 3A. Both pigments demonstrated strong absorbance in the UV region, with optical density gradually decreasing as the wavelength increased. This spectral pattern is consistent with the characteristic absorption profile of eumelanin, typically exhibiting maximum absorbance approximately 220 nm [28]. In this study, both pigments displayed distinct absorption maxima at approximately 211 nm (211.1 nm for the red variety and 210.8 nm for the yellow variety). These values are slightly blue-shifted compared to those reported for other fungal melanins: 215 nm for *A. auricula* [29], 220 nm for *M. sextelata* [19], 226 nm in *Inonotus hispidus* [30], and 235 nm for *Pleurotus* spp. [13]. This observation indicates that the pigments derived from the pileipellis of *S. rugosoannulata* belong to the melanin family, while exhibiting certain structural differences in monomer composition and conjugated systems compared to melanins from other fungal species.

#### 2.3.2. Fourier Transform Infrared (FTIR) Spectra

FTIR is the most widely employed spectroscopic technique for the investigation and characterization of melanin pigments, with a scanning range of 500–4000 cm^−1^ [28,31]. As illustrated in Figure 3B, the FTIR spectral profiles of the two isolated pigments demonstrated comparable trends, consistent with the previously reported similarities among melanins derived from three *Pleurotus* species [13]. Both pigments display a strong and broad characteristic absorption peak in the range of 3000–3500 cm^−1^, corresponding to the combined stretching vibrations of carboxyl, phenols, −OH and −NH_2_ groups, which are typically observed in eumelanin and pheomelanin [10,28]. The maximum absorption peak was observed at 3298 cm^−1^, closely resembling the melanins for *Pleurotus* species (3282 cm^−1^) and *M. sextelata* (3282 and 3008 cm^−1^) [13,19]. Furthermore, the pigments exhibited characteristic peaks at 2925 cm^−1^ attributed to C−H stretching (−CH_2_ and −CH_3_), 1712 cm^−1^ for C=O stretching, 1655 cm^−1^ for C=O or aromatic C=C stretching, 1540 cm^−1^ for N−H stretching, 1460 cm^−1^ for −CH_2_CH_3_ stretching, 1410 cm^−1^ for C−N stretching, 1241 cm^−1^ for phenolic −OH stretching, and 1147 cm^−1^ for COC stretching [10,28,32]. FTIR analysis indicates that the pigments from *S. rugosoannulata* exhibit characteristic absorption spectra similar to melanins of other fungi, including *A. auricula* [16], *Pleurotus* spp. [13], and *M. sextelata* [19]. The findings further corroborate that the two isolated pigments belong to the melanin family, while highlighting both shared characteristics and unique features among melanins derived from various fungal sources.

#### 2.3.3. Solid-State Nuclear Magnetic Resonance (NMR) Spectra

Owing to the inherent insolubility of melanins in aqueous solutions and their heterogeneous and amorphous nature, solid-state NMR spectroscopy is commonly employed to analyze the constituent chemical moieties of melanins [19,31]. As illustrated in Figure 3C, the ^13^C NMR spectra of the two isolated pigments revealed several characteristic peaks. The presence of peaks within the range of 160–180 ppm suggests the existence of carboxyl (COO) or amide (CONH) functional groups [10]. Peaks observed between 120 and 140 ppm were attributed to indolic or pyrrole-type carbons linked to hydrogen atoms or in an unprotonated state [32]. Additionally, signals appearing at 50–80 ppm were assigned to oxygenated aliphatic carbons (CHO and CH_2_O) [13,19], while peaks in the range of 10–40 ppm corresponded to carbon atoms from methyl and methylene groups [32,33]. Furthermore, peaks within 40–60 ppm were indicative of carbon atoms bonded to nitrogen or sulfur (C-N or C-S), which are characteristic features of pheomelanin [32,34]. Based on the combined FTIR and NMR spectral characteristics, both pigments contain functional groups such as phenolic hydroxyl, carboxyl, carbonyl, methylene, and methyl groups, which aligns with previously reported melanins from fungal species including *Pleurotus* spp. [13], *A. auricula* [16], and *M. sextelata* [19].

#### 2.3.4. Pyrolysis Gas Chromatography and Mass Spectrometry (Py-GCMS) Analysis

**Figure 4:**
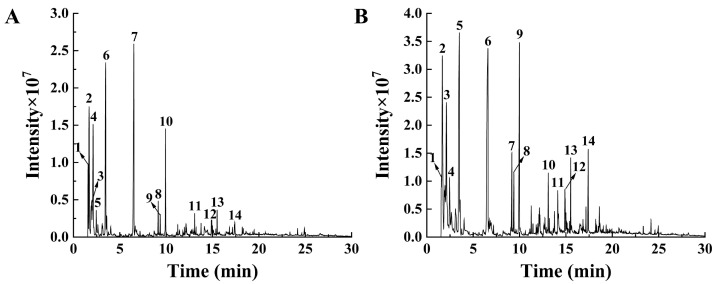
Py-GCMS profiles of isolated melanins from the red (**A**) and yellow (**B**) varieties of *S. rugosoannulata*. The numbers indicate the major pyrolysis products of melanins, which are detailed in Tables S1 and S2.

Py-GCMS is ideally suited for the structural characterization of melanin, as it provides monomer information and can distinguish between eumelanin and phaeomelanin from the pyrolysis products of complex compounds [20,35]. In this study, the thermal degradation profiles of the two isolated pigments are illustrated in Figure 4, with the major pyrolysis products detailed in Tables S1 and S2. The analysis detected the presence of toluene, styrene, indole, and certain benzene derivatives, as well as unsaturated olefin derivatives in the melanins of *S. rugosoannulata*. These compounds are recognized as characteristic markers of eumelanin thermal decomposition [36] and exhibit notable similarities to the melanins identified in the mushroom *Boletus griseus* [37] and *O. sinensis* [20]. Notably, toluene was identified as the predominant pyrolysis product in the melanin isolated from the red variety, whereas unsaturated olefin derivatives, azulene, indene, and indole were found to be significantly more abundant in the melanin derived from the yellow variety. These results highlight substantial compositional differences between the melanins of the red and yellow varieties of *S. rugosoannulata*, which aligns with previous reports on melanins from differently colored *Pleurotus* species [13] and *M. sextelata* [19]. Furthermore, in contrast to the pyrolysis products of melanins from *B. griseus* and *O. sinensis* [20,37], the two isolated melanins exhibited a higher abundance of alkanes and alkenes, indicating a greater proportion of unsaturated bonds. In brief, the Py-GCMS analysis results confirm that the pigments isolated from the caps of the two *S. rugosoannulata* varieties are melanins, specifically consisting of both eumelanin and pheomelanin.

#### 2.3.5. Elemental Composition Analysis

The elemental composition analysis of the two isolated pigments was performed using an elemental analyzer, and the results were compared with melanins from other fungal species, as summarized in Table 1. The pigment obtained from red variety consisted of 55.63% C, 7.40% H, 30.23% O, 5.99% N, and 0.64% S, whereas the pigment from the yellow variety comprised 52.22% C, 6.74% H, 29.70% O, 5.91% N, and 0.99% S. Previous studies have reported that the carbon content of eumelanin and pheomelanin is 52.22% and 56.45%, respectively, whereas that of melanins derived from various fungal sources ranges from 44.29% to 56.38% (Table 1). These findings are consistent with the results of the present study. This further indicates that melanin is an important type of pigment in the cap of *S. rugosoannulata*.

Eumelanin is synthesized through the oxidation pathways of L-tyrosine or L-3,4-dihydroxyphenylalanine (L-DOPA), exhibiting high contents of C, H, N, and O but lacking S. In contrast, pheomelanin primarily arises from the further derivation and polymerization of cysteinyl DOPA, resulting in a higher sulfur content (9–12%) [10,38]. The S content of the two pigments was 0.64% and 0.99%, respectively, which falls between that of eumelanin (0.09%) and phaeomelanin (9.78%). This suggests that the melanins from *S. rugosoannulata* varieties may contain both eumelanin and phaeomelanin forms, with eumelanin being the predominant form. Furthermore, based on comprehensive HPLC and elemental composition analyses, along with previous reports and the structural units of eumelanin (Figure S1), we hypothesize that the structural unit of eumelanin in *S. rugosoannulata* can be represented as [C_18_(OR)_3_H_7_O_4_N_2_]_n_ [37,39].

According to previous review studies, eumelanin exhibits colors ranging from black to brown, while pheomelanin displays colors from red to yellow [10]. The differences in the proportions of these two pigments may account for the variations in cap coloration observed in the two varieties of *S. rugosoannulata*. This finding aligns with earlier research on pigment diversity in different *Pleurotus* species [13]. Additionally, previous studies have indicated that the S content of fungal melanins ranges from 0.55% to 12.36%, containing varying proportions of eumelanin and pheomelanin, which is consistent with the results of this study [34,40]. Furthermore, the C/H, C/O, and C/N ratios of the two isolated pigments were found to be similar to each other and closely matched those of *M. sextelata* with brown caps, *M. sextelata* with black caps [19], and *G. lucidum* [40], respectively. This indicates that the elemental composition of melanins from *S. rugosoannulata* is similar to that of previously reported fungal melanins, while also exhibiting unique characteristics.

**Table 1:** Comparative analysis of elemental composition of melanins from *S. rugosoannulata* and other fungi.

Source		Content (%)					Ratio		
		C	H	O	N	S	C/H	C/O	C/N
*S. rugosoannulata* (This study)	Red cap	55.63	7.40	30.23	5.99	0.64	7.52	1.84	9.29
	Yellow cap	52.22	6.74	29.70	5.91	0.99	7.75	1.76	8.84
Synthetic eumelanin [41]		56.45	3.15	31.82	8.49	0.09	9.55	2.37	7.76
Phaeomelanin [41]		46.24	4.46	30.16	9.36	9.78	12.10	2.04	5.76
*Auricularia auricula* [16]		44.29	4.63	41.65	8.49	0.94	6.57	1.06	5.22
*Boletus griseus* [37]		56.38	5.86	28.04	6.17	2.44	9.62	2.01	9.13
*Ganoderma lucidum* [40]		54.20	6.24	33.35	5.14	1.07	8.69	1.63	10.54
*Morchella sextelata* [19]	Brown cap	49.99	6.85	35.18	7.27	0.71	7.30	1.42	6.88
	Black cap	53.55	5.84	30.10	9.28	1.23	9.17	1.78	5.77
*Ophiocordyceps sinensis* [20]		50.59	6.18	33.90	8.19	1.2	8.19	1.49	6.18
*Pleurotus cornucopiae* [13]	Black cap	52.96	7.37	28.03	11.09	0.55	7.19	1.89	4.78
*Pleurotus citrinopileatus* [13]	Yellow cap	50.51	7.09	31.79	9.78	0.83	7.12	1.59	5.16
*Pleurotus djamor* [13]	Pink cap	50.82	7.17	30.69	10.41	0.91	7.09	1.66	4.88
*Termitomyces albuminosus* [34]		54.68	3.54	26.92	2.49	12.36	15.43	2.03	21.94

### 2.4. Microscopic Analysis of the Pigments

#### 2.4.1. Scanning Electron Microscope (SEM) Analysis

**Figure 5:**
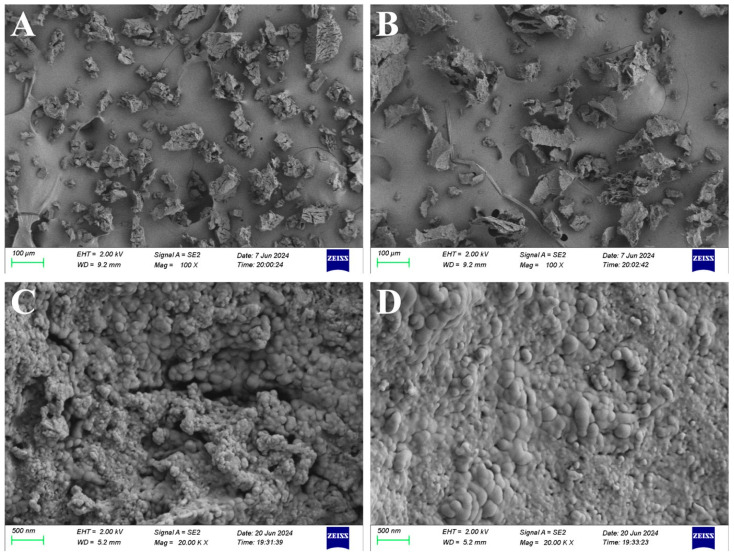
SEM images of the isolated melanins from *S. rugosoannulata* at two different magnifications. Images (**A**,**C**) depict the melanin from red variety, while images (**B**,**D**) represent the melanin from yellow variety. (**A**,**B**), scale bar = 100 μm; (**C**,**D**), scale bar = 500 nm.

The ultrastructural characteristics of the isolated melanins were characterized using SEM, as illustrated in Figure 5. The SEM micrographs revealed that both melanins consist of amorphous materials composed of numerous irregularly aggregated small granules, which are nearly spherical in shape and range in diameter from 50 to 200 nm. The observed ultrastructural features show remarkable consistency with previously reported characteristics of both synthetic and natural melanins, including artificially synthesized melanin [42], as well as melanins isolated from *A. auricula* [43], *Cryptococcus neoformans* [44], *M. sextelata* [19] and *Pleurotus* spp. [13]. This structural similarity across diverse melanin sources suggests a conserved nanoscale organization in melanin pigments regardless of their origin.

#### 2.4.2. Transmission Electron Microscopy (TEM) Analysis

**Figure 6:**
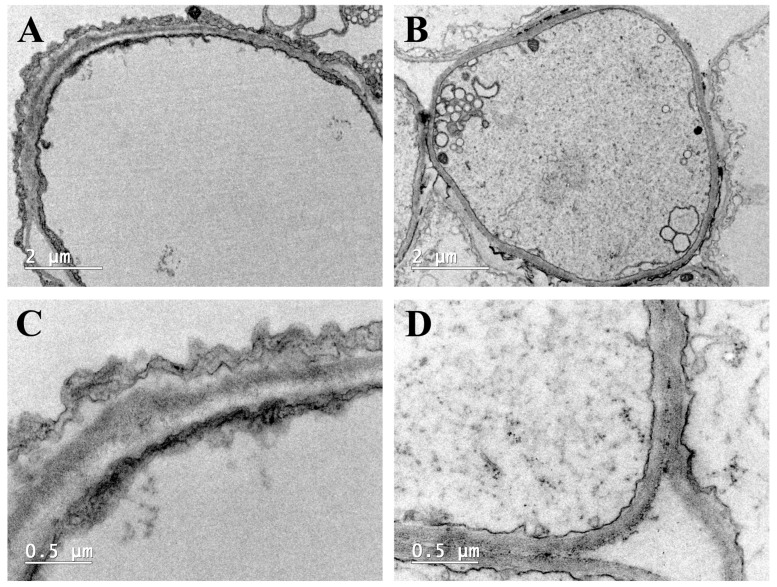
Ultrastructure under TEM of the pileipellis of *S. rugosoannulata* at two different magnifications. (**A**,**C**), red variety; (**B**,**D**), yellow variety; (**A**,**B**), scale bar = 2 μm; (**C**,**D**), scale bar = 0.5 μm.

Natural melanins are predominantly localized within the cell wall, exhibiting distinct electron-dense characteristics observable as dark regions in TEM micrographs [39,43]. The TEM micrographs revealed that melanins were primarily localized within the cell wall, with a higher concentration on the inner side compared to the outer side (Figure 6). Moreover, comparative ultrastructural analysis demonstrated a significantly higher accumulation of electron-dense granules along the inner cell wall of cap epidermal cells in the red variety compared to its yellow counterpart. This differential distribution pattern of melanin deposition is consistent with the distinct melanin localization characteristics observed in *M. sextelata* with black and brown caps [19]. Previous studies have indicated that *C. neoformans* synthesizes melanin via the L-DOPA pathway, which is localized in the innermost layer of the cell wall adjacent to the plasma membrane [45]. Furthermore, blue light can activate the L-DOPA pathway in *M. sextelata*, promoting melanin synthesis that significantly accumulates in the inner layer of the cell wall, leading to a marked darkening of the cap color in ascomata [19].

## 3. Materials and Methods

### 3.1. Mushroom Samples

The strains utilized in this study were collected from our laboratory, designated as SR-24-HNR (red caps) and SR-24-HWY (yellow caps) (Figure 1). Both strains were cultivated under standardized conditions at a commercial mushroom production facility located in Shunyi District, Beijing, China. Near-mature (not yet opened) fruiting bodies possess optimal commercial traits and rich pigments, and therefore, they were used for pigment isolation in this study. After harvesting, pileipellis tissues from typical red and yellow caps were aseptically collected and immediately flash-frozen in liquid nitrogen. Subsequently, the tissues were ground into fine powder using a pre-chilled mortar and pestle to prevent pigment degradation. The resulting homogenized powder was stored at −80 °C for subsequent extraction and analysis. Additionally, fresh harvested fruiting bodies were processed for ultrastructural examination using transmission electron microscopy (TEM) to investigate cellular pigment distribution and morphology.

### 3.2. Pigments Isolation and Purification

The pigments were isolated using an alkaline extraction and acid precipitation method as described by Zhang et al. [13], with further modifications. Specifically, the homogenized powder of pileipellis tissues was suspended in a 1.5 mol/L NaOH solution at a solid-to-liquid ratio of 1:25 (*w*/*v*). The suspension was then extracted using an ultrasonic extractor at 80 W and 60 °C for 2 h, followed by centrifugation at 8000 rpm for 20 min. The resulting supernatant was acidified to pH 1.5 using 7.0 mol/L HCl and incubated at room temperature for 3 h to facilitate complete pigment precipitation. After another centrifugation at 8000 rpm for 20 min, the precipitate was collected and washed repeatedly with distilled water until a neutral pH was achieved. Finally, the crude pigment samples were freeze-dried in a vacuum freeze dryer.

Subsequently, the crude pigment samples were sequentially washed with ethanol, chloroform, and ethyl acetate to remove sugars, proteins, and lipids, respectively [16]. Following air-drying at room temperature, the samples were dissolved in 50 mL of 1.5 mol/L NaOH solution and centrifuged at 8000 rpm for 20 min. The supernatant was collected and acidified to pH 1.5 using 2.0 mol/L HCl to precipitate the pigments. The resulting pigment precipitate was then collected by another centrifugation at 8000 rpm for 20 min. This alkaline dissolution and acid precipitation cycle was repeated four times to ensure purification efficiency. Finally, the precipitate was washed with distilled water until neutral and then freeze-dried to obtain the purified samples.

### 3.3. HPLC Analysis

The obtained pigment samples were analyzed using an Agilent 1290 HPLC system (Agilent, Santa Clara, CA, USA), with a commercially available eumelanin standard (M8631, Sigma-Aldrich, Burlington, MA, USA) serving as the reference. Prior to analysis, both the purified pigment samples and the eumelanin standard were dissolved in a 0.5 mol/L NaOH solution to achieve a final concentration of 100 mg/L. Chromatographic separation was performed on a Waters C_18_ column (300 mm × 7.8 mm, 5 μm particle size, Waters, Milford, MA, USA). The mobile phase, composed of 1% acetic acid (pH 2.8) and methanol in a volume ratio of 97:3, was delivered at a constant flow rate of 0.2 mL/min. The injection volume was set at 20 μL, and detection was conducted at a wavelength of 280 nm. The interval for the elution and conditioning column during each run was 30 min. To ensure reproducibility and optimal separation efficiency, the column temperature was maintained at 25 °C throughout the analysis [19,29].

### 3.4. Chemical Composition Analysis of the Pigments

#### 3.4.1. UV–Visible Light Absorption Spectra

The purified pigment samples were dissolved in 0.1 mol/L NaOH solution to achieve a final concentration of 200 mg/L. The spectral characteristics of the pigment solutions were analyzed using a UV–visible spectrophotometer (NanoBio 200, OPTOSKY, Xiamen, China) across the wavelength range of 200–700 nm, with 0.1 mol/L NaOH solution serving as the blank reference [19,20].

#### 3.4.2. FTIR Spectroscopy Analysis

FTIR spectroscopy analysis of the purified pigment samples was performed using a Nicolet iS10 spectrometer (Thermo Scientific, Waltham, MA, USA). Sample preparation was carried out following established protocols with modifications [13]. Briefly, the pigment samples were homogenously mixed with spectroscopic-grade potassium bromide (KBr) at a ratio of 1:20 (*w*/*w*) using an agate mortar. Approximately 30 mg of the homogeneous mixture was transferred into a stainless steel sample cup (10 mm diameter × 0.5 mm depth). The sample-loaded cup was then placed in the spectrometer’s reaction chamber under controlled atmospheric conditions. Spectral acquisition was performed using OMNIC software (version 8.3, Thermo Scientific) with the following parameters: spectral range of 4000–650 cm^−1^, a resolution of 4 cm^−1^, and 128 cumulative scans per spectrum to ensure an optimal signal-to-noise ratio. Prior to sample measurement, background spectra were collected using pure KBr pellets under identical instrumental conditions and were automatically subtracted from the sample spectra. All measurements were conducted in triplicate.

#### 3.4.3. Solid-State NMR Spectroscopy Analysis

Solid-state ^13^C cross-polarization magic angle spinning (CP/MAS) NMR experiments were conducted using a Bruker Avance III 600 NHz spectrometer (Bruker, Bremen, Germany), following established protocols [13].

#### 3.4.4. Py-GCMS Analysis

The structural composition of the purified pigment samples was characterized using Py-GCMS with a GCMS-QP2010 Ultra system (Shimadzu, Kyoto, Japan) [46]. The samples were subjected to pyrolysis at 770 °C for 8 s using a thermal pyrolyzer (EGA/PY-3030D, Frontier Laboratories, Tokyo, Japan). The injection port temperature was maintained at 220 °C, and with ultra-high-purity helium (99.999%) as the carrier gas, flowing at a rate of 1.8 mL/min under a constant pressure of 100 kPa. The pyrolytic products were separated using an Rtx-5MS fused silica capillary column (60 m × 0.32 mm i.d., 0.5 µm film thickness; Restek, Bellefonte, PA, USA), with the stationary phase consisting of 5% diphenyl and 95% dimethyl polysiloxane. The temperature programming for the chromatographic column was as follows: an initial temperature of 30 °C was held for 5 min, followed by a ramp to 260 °C at a rate of 10 °C/min, maintained for 16 min at 260 °C. The mass spectrometer operated in electron impact ionization (EI) mode, with an ionization energy of 70 eV and a full scan range of 45–450 m/z. The ion source temperature was set to 170 °C, while the quadrupole analyzer was maintained at 100 °C. Data acquisition and processing were performed using LabSolutions GCMS software (Shimadzu, version 5.92). To ensure experimental reproducibility, all samples were analyzed in triplicate, and the system was calibrated prior to the experiments using a standard alkane mixture (C7–C40) [20].

#### 3.4.5. Elemental Composition Analysis

Prior to elemental analysis, the purified pigment samples were dried in an oven at 60 °C for 1 h. Subsequently, the elemental composition, including carbon (C), hydrogen (H), oxygen (O), nitrogen (N), and sulfur (S), was quantitatively determined using an elemental analyzer (vario El cube, Elementar, Langenselbold, Germany) [19].

### 3.5. Microscopic Observation of the Pigments

#### 3.5.1. SEM

The ultrastructure of the purified pigment samples was characterized using SEM. Sample preparation followed the protocol previously described by Zhang et al. [13]. SEM observations were conducted using a SIGMA-500 field-emission scanning electron microscope (Carl Zeiss, Oberkochen, Germany) at an acceleration voltage of 2 kV.

#### 3.5.2. TEM

The ultrastructural characteristics of the pigments within the pileipellis of the two *S. rugosoannulata* varieties were examined using a transmission electron microscope (JEM1200EX, Tokyo, Japan). The sample pretreatment was performed according to the method described by Yan et al. [18]. In detail, the pileipellis samples were soaked in 2.5% (*v*/*v*) glutaraldehyde solution over night at 4 °C. They were then washed three times with phosphate-buffered saline (PBS, 0.1 mol/L, pH 7.0), with each wash lasting 15 min to remove any residual glutaraldehyde. Subsequently, the samples underwent secondary fixation with a 1% osmium tetroxide solution for 2 h, followed by another three washes with PBS, each lasting 15 min. Next, the samples were dehydrated sequentially with gradient ethanol solutions (30%, 50%, 70%, 80%, 90%, and 100%), with each step lasting 15 min. This was followed by treatment with anhydrous acetone for 20 min to achieve complete dehydration. The dehydrated samples were infiltrated with different volume ratios of acetone and epoxy resin (3:1, 1:1, and 1:3) for 2 h, 3 h, and 3 h, respectively. Subsequently, the samples were infiltrated with pure resin overnight. The resin-embedded samples were then heated at 35 °C, 60 °C, and 80 °C for 5 h each to complete the curing process and form hard resin blocks. Subsequently, the samples were sliced into ultrathin sections with a thickness of 70–90 nm using a LEICA EM UC7 ultramicrotome (Wetzlar, Germany). After sectioning, the slices were stained with uranyl acetate for 15 min, followed by staining with lead citrate for 5 min. Finally, the stained sections were air-dried and placed in the TEM for observation.

## 4. Conclusions

In summary, melanin pigments from the red and yellow varieties of *S. rugosoannulata* were successfully extracted and purified using an alkaline extraction and acid precipitation method. Both melanins exhibited a maximum absorption peak at approximately 211 nm. Elemental and Py-GCMS analyses revealed the presence of eumelanin and phaeomelanin, with eumelanin as the predominant form. The variations in the quantities and relative proportions of these melanins accounted for the observed color differences in the mushroom caps. Additionally, ultrastructural micrographs indicated that melanins were primarily localized within the cell wall, with a higher concentration on the inner side compared to the outer side. These findings provide valuable insights into the fundamental properties and potential applications of mushroom pigments. Melanin demonstrates strong antioxidant activity, and in practical production, the melanin content in fruiting bodies can be enhanced by optimizing management practices. Furthermore, tyrosinase is a crucial enzyme in the melanin synthesis pathway, and future research should concentrate on the regulation of melanin synthesis.

## Data Availability

Data will be made available on request.

## Supplementary Materials

The following supporting information can be downloaded at: https://www.mdpi.com/article/10.3390/ijms26146985/s1.
