# Supplementary material for: Isolation and Structural Characterization of Melanins from Red and Yellow Varieties of Stropharia rugosoannulata
Source: Int J Mol Sci. 2025 Jul 21;26(14):6985. doi: 10.3390/ijms26146985 (PMC12295496; doi:10.3390/ijms26146985)
Supplement: Supplementary file 1 [file ijms-26-06985-s001.zip › ijms-3730019-supplementary.pdf]

Isolation and structural characterization of pigments from red and yellow varieties of *Stropharia rugosoannulata*

Zhen-Fei Xie[a, 1], Wei-Wei Zhang[a, 1], Shun-Yin Zhao[a], Xiao-Han Zhang[a], Shu-Ning You[a], Chun-Mei Liu[b, *], Guo-Qing Zhang[a, *]

[a] College of Plant Science and Technology, Beijing University of Agriculture, Beijing 102206, China

[b] College of Bioscience and Resources Environment, Beijing University of Agriculture, Beijing 102206, China

[1] These authors contributed equally to this work.

* Corresponding authors.

E-mail: zhanggqbua@163.com (Guo-Qing Zhang)

15910413793@163.com (Chun-Mei Liu)

Supplementary data

Table S1. The Py-GCMS products of the melanin from isolated the red variety of *S. rugosoannulata*.

Num.	Ret. Time (min)	Area (%)	Height	Name	Structure
1	1.592	34686634	9750375	L-Lactic acid	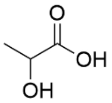
2	1.686	70648049	17458364	1,3-Butadiene	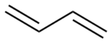
3	1.959	19218965	4639172	1,3-Pentadiene	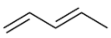
4	2.111	58128275	14944758	1-Penten-3-yne	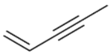
5	2.456	11129653	3271939	1-Hexene	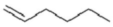
6	3.469	85859784	23241690	1,3-Hexadien-5-yne	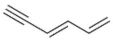
7	6.519	115675601	25772858	Toluene	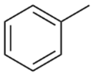
8	9.138	10088756	4675251	Ethylbenzene	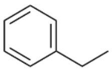
9	9.361	6111343	2862438	Benzene, 1,3-dimethyl-	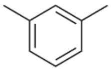
10	9.933	32886132	14331190	Styrene	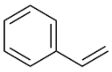
11	13.075	6430678	2951108	Indene	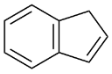
12	14.876	4786216	2063907	2-Methylindene	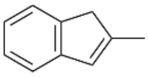
13	15.492	6912811	3372534	Azulene	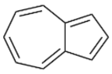
14	17.355	2906482	1220212	Indole	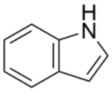

Num.: Number; Ret. Time: Retention Time.

Table S2. The Py-GCMS products of the melanin from isolated the yellow variety of *S. rugosoannulata*.

Num.	Ret. Time (min)	Area (%)	Height	Name	Structure
1	1.574	29789039	10945581	4-Pentyn-2-ol	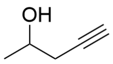
2	1.674	172658018	32430469	Bicyclo [1.1.0] butane	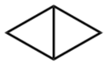
3	2.107	160306909	23999175	Cyclopropylacetylene	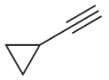
4	2.456	55000958	10347369	1-Hexene	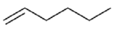
5	3.5	222244463	36267960	1,5-Hexadiyne	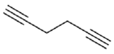
6	6.593	285316581	33428555	1,5-Heptadien-3-yne	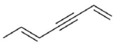
7	9.162	37668691	14642583	Ethylbenzene	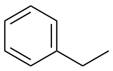
8	9.386	29237179	11080566	Benzene, 1,3-dimethyl-	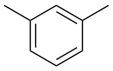
9	9.976	109666249	34315657	1,3,5,7-Cyclooctatetraene	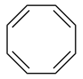
10	13.098	26208995	11163767	1-Chloroindan	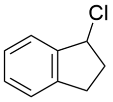
11	14.122	28804736	7965348	Phenol	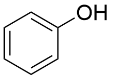
12	14.892	34626571	7862953	Indene	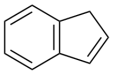
13	15.521	29388884	12642375	Azulene	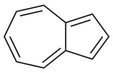
14	17.4	42969178	14036328	Indole	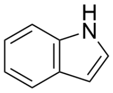

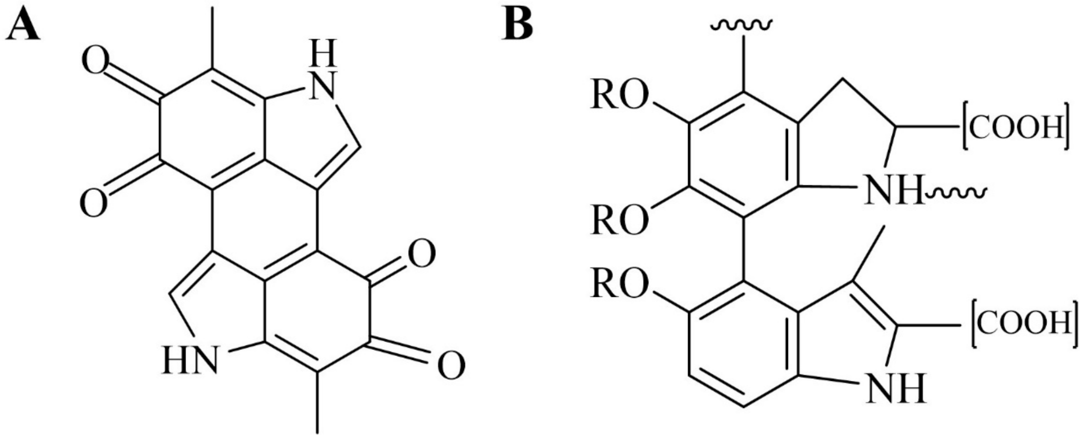

Figure S1. Structural unit of standard eumelanin (A) and proposed eumelanin from *S. rugosoannulata* (B). R = H / fatty acid / sugar / amino acid.
